# Antigenic and Molecular Genetic Evolution of Influenza Viruses in Kazakhstan from 2017 to 2025

**DOI:** 10.3390/biology15181640

**Published:** 2026-09-16

**Authors:** Altynay Gabiden, Aknur Mutaliyeva, Aidar Usserbayev, Assem Yermekbayeva, Azamat Kenessov, Meyramgul Smagulova, Andrey Komissarov, Alexander Perederiy, Artem Fadeev, Kirill Stolyarov, Askar Abdaliyev, Dariya Jussupova, Lena Kassabekova, Meirim Sultanova, Ainagul Kuatbaeva, Manar Smagul

**Affiliations:** 1 National Center of Public Health Care of the Ministry of Health of the Republic of Kazakhstan, Almaty 050000, Kazakhstan; 2 Smorodintsev Research Institute of Influenza, Saint Petersburg 190000, Russia; 3 National Center of Expertise of the Committee of Sanitary and Epidemiological Control of the Ministry of Health of the Republic of Kazakhstan, Astana 010000, Kazakhstan; 4 Department of Natural Sciences and Geography, Abai Kazakh National Pedagogical University, Almaty 050000, Kazakhstan

**Keywords:** influenza, antigenic characteristics, virus evolution, mutation, subclade, phenotypic characteristics, next-generation sequencing (NGS), nanopore sequencing, Kazakhstan

## Abstract

Influenza viruses undergo continuous genetic and antigenic evolution, making sustained surveillance essential for monitoring changes in virus circulation and vaccine relevance. We analyzed influenza epidemiology and viral evolution in Kazakhstan across eight consecutive epidemic seasons (2017/18–2024/25) using data from the national influenza sentinel surveillance system, together with antigenic characterization, Sanger sequencing, whole-genome sequencing, and phylogenetic analysis. Influenza activity was almost undetectable during the 2020/21 season, followed by the re-emergence of A(H3N2)-dominated circulation in 2021/22. In 2022/23, influenza B predominated initially, followed by A(H1N1)pdm09, whereas A(H3N2) predominated in 2023/24. A total of 199 A(H1N1)pdm09, 157 A(H3N2), 105 B/Victoria, and 16 B/Yamagata viruses were genetically characterized. A(H1N1)pdm09 viruses showed successive replacement of 6B-derived lineages, with descendants of 6B.1A.5a.2a predominating in the post-pandemic period. A(H3N2) viruses showed substantial genetic diversification, progressing from 3C.2a descendants to 3C.2a1b-related viruses and subsequently to 2a.3a.1/J.2 viruses. Among B/Victoria viruses, V1A was successively replaced by V1A.3 and V1A.3a.2, with V1A.3a.2 accounting for 100% of genetically characterized viruses in 2023/24 and 2024/25. Antigenic analysis showed 16- to 32-fold lower HI titers for A(H1N1)pdm09 6B.1A.5a.2 viruses when tested against antisera to earlier vaccine/reference strains, whereas no detectable antigenic differences were observed between 6B.1A.5a.2a and its recent descendants. These findings demonstrate substantial post-pandemic changes in influenza epidemic timing, subtype dominance, and genetic composition in Kazakhstan, while circulating viruses remained within globally recognized evolutionary lineages. Integrated epidemiological, genetic, and antigenic surveillance is important for detecting emerging variants, assessing vaccine relevance, and supporting timely influenza prevention and control measures.

## Simple Summary

Influenza viruses continuously evolve, which can change their circulation patterns and reduce recognition by existing immunity and vaccine-induced antibodies. Continuous surveillance is therefore important for monitoring circulating viruses and informing influenza prevention and vaccine policy. In this study, we analyzed influenza viruses circulating in Kazakhstan over eight consecutive epidemic seasons (2017/18–2024/25) using data from the national influenza sentinel surveillance system, together with epidemiological, antigenic, molecular genetic, and phylogenetic analyses. We observed substantial changes in influenza circulation following the COVID-19 pandemic. Influenza activity was almost undetectable during the 2020/21 season, followed by A(H3N2)-dominated circulation in 2021/22. In 2022/23, influenza B predominated initially, followed by A(H1N1)pdm09, while A(H3N2) predominated in 2023/24. Genetic analysis identified 199 A(H1N1)pdm09, 157 A(H3N2), 105 B/Victoria, and 16 B/Yamagata viruses. We observed successive replacement of major genetic lineages within A(H1N1)pdm09, A(H3N2), and B/Victoria viruses. Antigenic analysis showed a 16- to 32-fold reduction in HI titers of A(H1N1)pdm09 6B.1A.5a.2 viruses when tested against antisera to earlier vaccine/reference strains. These findings demonstrate substantial changes in the timing, subtype dominance, and genetic composition of influenza viruses in Kazakhstan following the COVID-19 pandemic. Continuous integrated epidemiological, genetic, and antigenic surveillance is important for detecting changes in circulating viruses, assessing vaccine relevance, and supporting national influenza prevention and control strategies.

## 1. Introduction

Influenza viruses remain one of the leading causes of acute respiratory infections worldwide, causing annual seasonal epidemics and contributing substantially to global morbidity and mortality [1,2]. According to international estimates, seasonal influenza results in millions of severe cases and hundreds of thousands of deaths each year, highlighting its significant public health burden [1]. The sustained epidemic potential of influenza viruses is primarily driven by their pronounced antigenic and genetic variability, which enables them to evade population immunity and maintain continuous circulation [2,3].

A central role in influenza virus evolution is played by the surface glycoproteins hemagglutinin (HA) and neuraminidase (NA), which represent the main antigenic determinants of the virus [3,4]. Antigenic drift, resulting from the accumulation of point mutations in HA and NA genes, leads to a gradual decline in population immunity and necessitates regular updates of vaccine strain composition [5]. In addition, the segmented nature of the influenza genome facilitates reassortment events, contributing to the emergence of novel genetic variants with altered biological properties [6].

Recent molecular studies have demonstrated that influenza virus evolution is shaped by a complex interplay between mutation processes, host immune pressure, and global human mobility [7,8]. In recent years, influenza A(H1N1)pdm09, A(H3N2), and influenza B viruses (Victoria and Yamagata lineages) have predominated globally, exhibiting distinct evolutionary dynamics and antigenic profiles [9,10]. Notably, A(H3N2) viruses show rapid antigenic drift driven by mutations in key antigenic sites and changes in glycosylation patterns, whereas A(H1N1)pdm09 viruses display comparatively more stable phylogenetic dynamics [10,11]. Influenza B viruses have also undergone notable changes, including shifts in lineage circulation and a marked decline in the detection of the B/Yamagata lineage following the COVID-19 pandemic [12].

Advances in whole-genome sequencing technologies have significantly enhanced the understanding of influenza virus evolution by enabling detailed analysis of genetic diversity, phylogenetic relationships, and mutations associated with antigenic variation and antiviral resistance [13,14]. The integration of genomic, antigenic, and epidemiological data has become a cornerstone of modern influenza surveillance, particularly within the framework of the World Health Organization’s Global Influenza Surveillance and Response System (GISRS) [5].

Kazakhstan, located at the crossroads of major migration routes in Eurasia and characterized by pronounced seasonal climatic conditions, represents an important region for studying influenza virus circulation and evolution. The national epidemiological and virological surveillance system, integrated into global monitoring networks, provides continuous data on circulating strains and their molecular characteristics. Previous studies have shown that influenza viruses circulating in Kazakhstan largely correspond to globally circulating genetic clades; however, the presence of unique amino acid substitutions suggests ongoing local evolutionary processes [15,16].

The period from 2017 to 2024 is of particular interest, as it encompasses pre-pandemic, pandemic, and post-pandemic phases associated with the COVID-19 crisis, which significantly altered the epidemiology of respiratory viruses [17]. Public health interventions implemented during the pandemic led to a marked reduction in influenza activity in 2020/21, followed by a gradual resurgence accompanied by shifts in circulating variants and their genetic composition [12,18].

Previous studies have provided important epidemiological, serological, and molecular surveillance data on influenza and other respiratory viruses in Kazakhstan [15,16]. However, these studies primarily addressed respiratory virus circulation, recent influenza seasons, or molecular characterization of selected influenza viruses. A longitudinal analysis integrating epidemiological patterns with clade and lineage dynamics, phylogenetic relationships, and HI-based antigenic characterization across the pre-pandemic, pandemic-disrupted, and post-pandemic periods remains limited. In particular, the temporal relationship between changes in influenza subtype and lineage dominance, genetic diversification, and antigenic evolution across multiple epidemic seasons has not been systematically examined within a single analytical framework.

In particular, there is a lack of detailed understanding of the genetic diversity and phylogenetic relationships of influenza A(H1N1)pdm09, A(H3N2), and B viruses, their antigenic evolution in relation to vaccine strains, and their molecular features, including potential markers associated with antiviral resistance.

The aim of this study was to characterize the epidemiological dynamics, antigenic properties, molecular evolution, and phylogenetic diversity of influenza viruses circulating in Kazakhstan during the 2017/18–2024/25 epidemic seasons, including genetic and antigenic diversification and potential molecular markers associated with antiviral resistance. The findings of this study are expected to enhance understanding of regional viral evolution and contribute to the optimization of influenza surveillance and vaccine strategies.

## 2. Materials and Methods

### 2.1. Epidemiological Surveillance System and Sample Collection

The study was conducted within the framework of the national influenza sentinel surveillance system in the Republic of Kazakhstan. Sentinel surveillance is implemented through a network of selected healthcare facilities that systematically collect clinical specimens from patients meeting the case definitions for influenza-like illness (ILI) and severe acute respiratory infection (SARI), in accordance with World Health Organization (WHO) guidelines. Routine surveillance includes laboratory diagnosis of respiratory infections performed as part of standard clinical practice across all regions of the country.

Primary clinical specimens (nasopharyngeal and/or oropharyngeal swabs) were collected at healthcare facilities and transported to 18 regional virology laboratories, where initial molecular testing was performed.

Influenza-positive samples identified through sentinel surveillance were subsequently referred to the Reference Laboratory for Viral Infection Control (Almaty), which serves as the National Influenza Center of Kazakhstan, for further antigenic, molecular genetic, and phylogenetic characterization.

### 2.2. Epidemiological Data Analysis

A retrospective analysis of influenza epidemics over eight consecutive seasons (2017/18–2024/25) was conducted, covering epidemiological weeks 40 through 39 of each season. The analysis was based on data obtained from the sentinel surveillance system for influenza-like illness (ILI). ILI was defined as a case of acute respiratory illness presenting with cough and a measured body temperature of ≥38 °C, with symptom onset within 7 days before May 2021 and within 10 days thereafter, following a revision of the local sentinel surveillance guideline [19,20].

Data were extracted from the national electronic surveillance system (data management system “Monitoring of ARVI/Influenza/Pneumonia”, https://ilisari.kz/ (accessed on 25 July 2026)) used in the Republic of Kazakhstan for the management of sentinel epidemiological surveillance data. Weekly aggregated datasets were retrieved, including epidemiological indicators as well as laboratory testing results for influenza virus detection performed by real-time polymerase chain reaction (RT-PCR).

Data processing and statistical analysis were performed using Microsoft Excel and StatSoft Statistica 10 software. Differences between proportions were calculated using Fisher’s z-test; the Benjamini–Hochberg procedure was applied to control the false discovery rate (FDR) at 5% within each table (140 pairwise comparisons across seasons and age groups for Table 1; 105 for Table 2), with statistical significance determined at adjusted *p* < 0.05.

### 2.3. Molecular Detection, Typing, and Subtyping of Influenza Viruses

Viral RNA extraction from respiratory clinical specimens was performed in regional laboratories using commercial kits “Ribo-sorb” and “Ribo-prep” (Central Research Institute of Epidemiology, Rospotrebnadzor, Russia), according to the manufacturers’ instructions.

Detection of influenza viruses was carried out using real-time reverse transcription polymerase chain reaction (RT-PCR) with the AmpliSens^®^ Influenza virus A/B-FL assay. Typing and subtyping of influenza A viruses were performed using AmpliSens^®^ Influenza virus A-type-FL and AmpliSens^®^ Influenza virus A/H1-swine-FL kits.

Identification of influenza B viruses and determination of their lineage (Victoria or Yamagata) were performed at the National Influenza Center using WHO-recommended RT-PCR protocols, including the CDC Influenza SARS-CoV-2 Multiplex Assay.

### 2.4. Virus Isolation in Cell Culture and Antigenic Analysis

For antigenic characterization, influenza viruses were isolated in Madin–Darby Canine Kidney (MDCK) cell culture, recommended by WHO for influenza virus propagation.

Clinical specimens confirmed to be influenza-positive and with sufficient viral load (Ct ≤ 28–30) were inoculated onto MDCK cell monolayers maintained in Minimum Essential Medium (MEM) supplemented with antibiotics and trypsin, following WHO standard protocols. Infected cultures were incubated at 33–35 °C and monitored daily for cytopathic effect (CPE).

The presence of virus in culture supernatants was confirmed by hemagglutination assay (HA) using 0.75% human group O erythrocytes. Isolated viruses were used for further antigenic analysis.

Antigenic characterization of influenza A and B viruses was performed by hemagglutination inhibition (HI) assay using post-infection ferret antisera raised against vaccine and reference strains. Turkey red blood cells (0.7%, *v*/*v*) were used for A(H1N1)pdm09 and influenza B viruses. A(H3N2) viruses were tested using guinea pig red blood cells in the presence of 20 nM oseltamivir to suppress neuraminidase-mediated binding. Antigenic reactivity was assessed by comparing HI titers with the homologous titer of the corresponding reference virus.

### 2.5. Sequencing of Influenza Viruses

Samples for sequencing were selected based on sufficient viral load (Ct ≤ 30).

Viral RNA extraction was performed using QIAamp Viral RNA Mini Kit, QIAGEN, Hilden, Germany)) and PureLink RNA Mini Kit (Thermo Fisher Scientific, Waltham, MA, USA), according to the manufacturers’ protocols.

#### 2.5.1. Sanger Sequencing

Amplification of HA and NA gene fragments was performed using RT-PCR with original primers (London protocol) and SuperScript^®^ III One-Step RT-PCR System with Platinum^®^ Taq DNA Polymerase (Life Technologies, Carlsbad, CA, USA) on a Veriti thermal cycler (Applied Biosystems, Foster City, CA, USA).

DNA electrophoresis was carried out using the “EF-200” agarose gel electrophoresis system (AmpliSens, Moscow, Russia) in 1.7% agarose gel with Tris-borate buffer and ethidium bromide. DNA fragments were purified using QIAquick Gel Extraction Kit (QIAGEN, Hilden, Germany) or PureLink PCR Purification Kit (Life Technologies, Carlsbad, CA, USA), according to the manufacturer’s instructions.

Capillary sequencing was performed on an Applied Biosystems 3500 Genetic Analyzer (Thermo Fisher Scientific, Waltham, MA, USA) using the BigDye Terminator v3.1 Cycle Sequencing Kit (Applied Biosystems, Foster City, CA, USA). Sequencing products were purified using BigDye XTerminator Kit (Applied Biosystems, Foster City, CA, USA).

#### 2.5.2. Whole-Genome Sequencing

Whole-genome sequencing of influenza viruses was performed using two next-generation sequencing platforms:

Illumina MiniSeq (Illumina, San Diego, CA, USA). cDNA synthesis and genome amplification were carried out using a multisegment RT-PCR approach. Library preparation was performed using AmpliSeq Library PLUS for Illumina kits (Illumina, San Diego, CA, USA). Library purification was conducted using AMPure XP magnetic beads (Beckman Coulter, Brea, CA, USA). Library quality and fragment size distribution were assessed using Agilent High Sensitivity DNA Kit on a Bioanalyzer 2100 (Agilent Technologies, Santa Clara, CA, USA). Sequencing was performed in paired-end mode using MiniSeq High Output reagent kits (Illumina, San Diego, CA, USA) according to the manufacturer’s instructions.

Oxford Nanopore Technologies. Selected samples were sequenced using Oxford Nanopore platforms (MinION; Oxford Nanopore Technologies, Oxford, UK. Genome amplification was performed using multisegment RT-PCR. Library preparation was carried out using Native Barcoding Kit 96 (Oxford Nanopore Technologies, Oxford, UK), NEBNext Ultra II End Repair/dA-Tailing Module (New England Biolabs, Ipswich, MA, USA), and NEBNext Quick Ligation Module, followed by sequencing using Flongle flow cells (Oxford Nanopore Technologies, Oxford, UK).

### 2.6. Bioinformatic and Phylogenetic Analysis

Quality assessment of Illumina sequencing data was performed using FastQC v0.13.0, and adapter and low-quality sequences with QV < 20 were removed using Fastp v1.3.6. Reads were aligned to the corresponding influenza reference genomes using BWA v0.7.19. Consensus sequences were generated using SAMtools mpileup v1.24 and iVar v1.4.4 with default consensus calling parameters (majority rule consensus, minimum QV = 20, minimum coverage depth = 10). For Oxford Nanopore sequencing data, basecalling and quality trimming were performed using Oxford Nanopore Dorado v2.1.2 with the high accuracy (hac@5.1.0) model with QV threshold = 10. Reads were aligned to reference genomes using Minimap2 v2.31, draft consensus sequences were obtained using iVar v1.4.4 with modified consensus calling parameters (majority rule consensus, minimum QV = 10, minimum coverage depth = 10) and final polished consensus sequences were generated using medaka_consensus script in Medaka v2.2.2. Only sequences with ≥90% genome coverage and a low proportion of ambiguous nucleotides were retained for downstream analyses.

Potential molecular markers associated with antiviral resistance were screened in the available viral genome sequences based on previously described resistance-associated substitutions. The identification of these molecular markers was interpreted as a genotypic indication only and was not considered evidence of confirmed antiviral resistance or susceptibility. Phenotypic antiviral susceptibility was not assessed in this study.

HA gene sequences and associated metadata were downloaded from GISAID for B/Yamagata (10,686 records), B/Victoria (58,965), A(H1N1)pdm09 (123,463), and A(H3N2) (143,735) viruses. Following quality control and removal of duplicate accession numbers, 10,684, 58,675, 122,778, and 142,959 unique accessions, respectively, were retained.

Exact deduplication of sequences sharing 100% nucleotide identity collapsed 3890, 26,308, 48,414, and 67,455 redundant accessions, resulting in 6794, 32,367, 74,364, and 75,504 unique sequence groups for B/Yamagata, B/Victoria, A(H1N1)pdm09, and A(H3N2), respectively. One representative sequence was selected from each group, while the identifiers of all identical sequences were retained in the FASTA header and separated by the “|” character.

Kazakhstan isolates were excluded from the background datasets, and the background sequences were clustered separately for each season using MMseqs2 v18.8cc5c [21], applying thresholds of 99% sequence identity and 95% coverage.

All unique Kazakhstan sequences were then used as queries against the seasonal representative datasets. For each Kazakhstan sequence–season combination, up to 10 of the closest background sequences with ≥99% identity were selected; when necessary, the dataset was supplemented with matches showing 98–99% identity. The selected background sequences, all Kazakhstan sequences, and the corresponding WHO-recommended vaccine strains were combined into the final datasets for subsequent phylogenetic analysis.

For each virus type, sequences from the resulting dataset were aligned independently using MAFFT. Maximum-likelihood phylogenetic trees were then inferred from the nucleotide alignments using RAxML-NG v2.0.2 under the GTR + G + FO nucleotide substitution model. The resulting phylogenetic trees were visualized in R v4.6.1 using the ggtree package v4.2.0.

Antigenic maps were constructed using Racmacs v.1.2.10 library in R (https://acorg.github.io/Racmacs/) (accessed on 10 July 2026). Antigenic distances are expressed in antigenic units (AU), with one grid unit corresponding to a two-fold difference in HI titer. Map orientation is arbitrary.

### 2.7. Ethical Considerations

The study was conducted within the framework of the national influenza sentinel surveillance system in the Republic of Kazakhstan. Clinical specimens were collected in accordance with the applicable national requirements for influenza surveillance and diagnostic activities. All data and specimens were anonymized prior to analysis, and no additional procedures beyond routine diagnostic and surveillance activities were performed for the purposes of this study.

The study protocol involving influenza and COVID-19 clinical specimens was reviewed and approved by the Local Bioethics Committee of the RSE on the Right of Economic Management “National Center for Public Health” of the Ministry of Health of the Republic of Kazakhstan (Protocol No. 1, dated 30 April 2021). This approval applies to the relevant research activities and materials covered from the date of approval onward and does not cover specimens collected prior to 30 April 2021. Earlier surveillance-derived specimens and data were collected within the established national influenza sentinel surveillance system and were not collected specifically for this study.

The ethical status of the earlier surveillance activities was further clarified by the written determination of the Local Ethics Committee dated 26 September 2023 (No. 10), which states that activities conducted as part of routine epidemiological surveillance for public health purposes are exempt from full ethical review and informed consent requirements under the applicable regulations of the Republic of Kazakhstan.

## 3. Results

### 3.1. Epidemiological Characteristics of Influenza Circulation in Kazakhstan, 2017/18–2024/25

**Figure 1:**
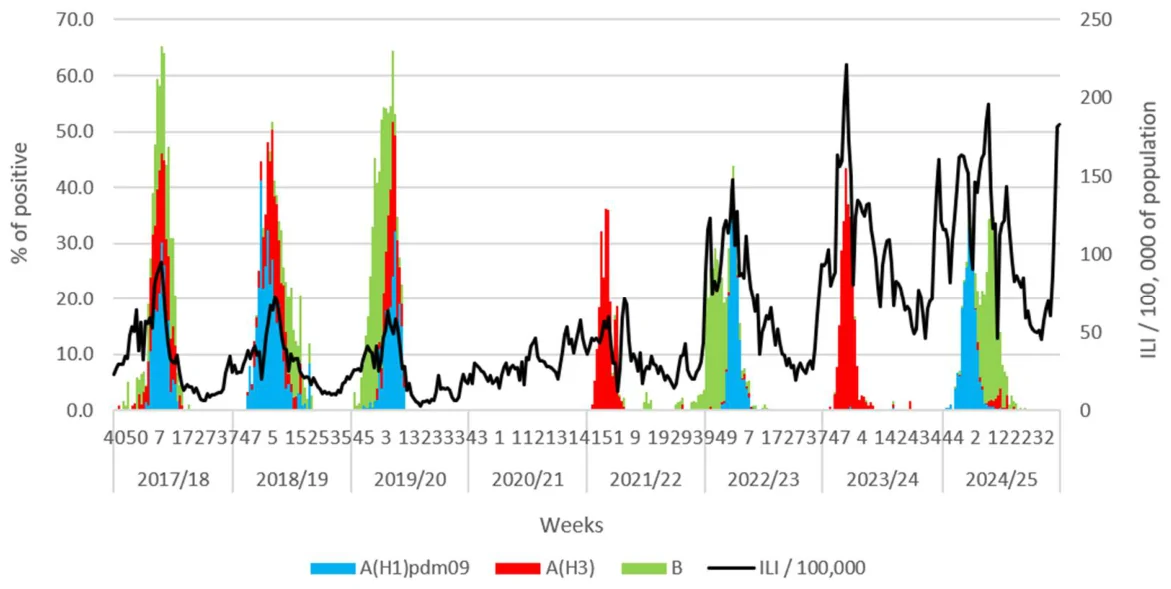
ILI activity and etiological profile of the influenza epidemics, 2017/18–2024/25.

The study period covered three epidemiologically distinct phases: the pre–COVID-19 period, the pandemic period (March 2020–May 2023), and the post-pandemic period. Each phase demonstrated specific trends in the development of the influenza epidemic process. The epidemiological dynamics and etiological profile of influenza circulation across the study period are shown in Figure 1.

During the 2017/18 season, all three influenza virus types—A(H1)pdm09, A(H3), and B—circulated simultaneously and in approximately equal proportions. Although the first influenza viruses were detected in December 2017, it did not substantially affect influenza-like illness (ILI) incidence. The epidemic increase in ILI incidence was registered in week 5 of 2018. The epidemic peak occurred in week 7 of 2018 (95.0 per 100,000 population), and the epidemic ended in week 14 of 2018. The total duration of the epidemic was 10 weeks.

The 2018/19 influenza epidemic began in week 50 of 2018 with circulation of influenza A(H1)pdm09. However, due to the New Year holidays, no sharp increase in ILI incidence was observed in 2018. The epidemic rise started in week 1 of 2019. From the same week, influenza A(H3) joined circulation, although its proportion remained lower than that of A(H1)pdm09. The epidemic peak occurred in week 6 of 2019 (72.6 per 100,000 population). Influenza B joined circulation in week 10 of 2019 but did not result in a significant increase in morbidity. Due to the sequential introduction of different influenza subtypes, the epidemic duration extended to 20 weeks.

The 2019/20 epidemic began considerably earlier, in week 47 of 2019, with the emergence of influenza B, which predominated until early 2020. Subsequently, both A(H1)pdm09 and A(H3) subtypes entered circulation simultaneously. The ILI peak occurred in week 4 of 2020 (63.2 per 100,000 population). In March 2020, the COVID-19 pandemic caused by SARS-CoV-2 was declared, placing substantial pressure on healthcare services and laboratories [22]. These factors resulted in a temporary disruption of sentinel surveillance starting from week 10 of 2020. Additionally, public health measures aimed at reducing interpersonal contact were implemented. Despite these factors, based on ILI incidence trends, the influenza epidemic can be considered to have ended in week 11 of 2020, with a total duration of 17 weeks.

The 2020/21 season was exceptional. Influenza circulation decreased by 99.8% compared with previous seasons [23]. Despite full restoration of influenza testing capacity, the sentinel surveillance system did not detect any laboratory-confirmed influenza cases.

In the 2021/22 season, SARS-CoV-2 continued to circulate actively; however, influenza virus re-emerged in October 2021 with the introduction of subtype A(H3). No significant increase in ILI incidence was observed, possibly due to ongoing quarantine measures. Based on laboratory data, the epidemic began in week 44 of 2021 and ended in week 1 of 2022, with a peak in week 48 of 2021 (59.7 per 100,000 population). The epidemic duration was 10 weeks. A(H3) was the dominant subtype. Sporadic influenza B cases were detected, while A(H1)pdm09 was not identified in any sentinel specimens.

The 2022/23 epidemic began unusually early, in week 40 of 2022, with influenza B circulation dominating until mid-December 2022. In late December 2022, A(H1)pdm09 emerged and rapidly became dominant, almost completely replacing influenza B. The epidemic peak occurred in week 52 of 2022 (147.5 per 100,000 population), and the epidemic ended in week 4 of 2023. The duration was 17 weeks. No A(H3) cases were detected within the sentinel surveillance system.

In the 2023/24 season, the epidemic also began relatively early, with the detection of A(H3) in week 46 of 2023, resulting in a substantial increase in ILI incidence. The peak occurred in week 50 of 2023 (220.9 per 100,000 population), and the epidemic ended in week 2 of 2024. The duration was 9 weeks. Similar to the 2021/22 season, A(H3) was overwhelmingly dominant, while A(H1)pdm09 and influenza B were detected only sporadically.

In the 2024/25 season, the epidemic began in week 47 of 2024. Initially, A(H1)pdm09 predominated and was associated with a significant increase in ILI incidence. By week 2 of 2025, A(H1)pdm09 circulation declined and influenza B became dominant, remaining so until the end of the epidemic. The peak occurred in week 8 of 2025 (195.9 per 100,000 population), and the epidemic ended in week 12 of 2025. The total duration was 18 weeks.

Analysis of influenza epidemics from 2017/18 to 2024/25 allows the following conclusions:

Despite the official COVID-19 pandemic period from March 2020 to May 2023, as well as the continued circulation of SARS-CoV-2 in subsequent years, only one season (2020/21) demonstrated substantial deviation from typical seasonal influenza epidemic patterns and occurred with virtually no influenza virus circulation.

The etiology of influenza epidemics before and after the 2020/21 season differs substantially. Before 2020/21, epidemics were characterized by annual co-circulation of all three influenza subtypes. After 2020/21, a distinct periodic pattern emerged: seasons with absolute dominance of A(H3) alternated annually with seasons characterized by sequential dominance of A(H1)pdm09 and influenza B (in varying order).

Post-2020/21 epidemics dominated by A(H3) were significantly shorter than those dominated by A(H1)pdm09 and B. However, this feature is not entirely unique to the post-pandemic period, as simultaneous circulation (2017/18) was also associated with shorter epidemic duration compared to seasons with sequential subtype circulation (2018/19 and 2019/20).

A direct comparison of ILI incidence before and after the COVID-19 pandemic is currently not feasible due to a modification of the ILI case definition within the sentinel surveillance system in May 2021. Previously, ILI was defined as symptom onset within ≤7 days; following alignment with the WHO standard case definition, this period was extended to 10 days. This change increased the number of cases meeting the ILI definition, resulting in higher reported ILI incidence in post-2020/21 seasons. Additionally, increased public awareness and healthcare-seeking behavior following the COVID-19 pandemic may have contributed to higher reporting of respiratory illnesses, particularly at the primary healthcare level [24,25]. The relative contribution of these factors remains uncertain and warrants further investigation.

### 3.2. Demographic Characteristics

Using weekly aggregated ILI surveillance data, the age distribution of cases was analyzed according to the following WHO-recommended sentinel age groups: 0–4, 5–14, 15–29, 30–64, and ≥65 years [20].

The proportion of ILI cases among children aged 0–4 years varied only slightly over the study period. The highest proportions were observed in 2017/18 (53.3%) and 2024/25 (50.5%), while the lowest were recorded in 2018/19 (36.3%) and during 2020/21 (38.7%) and 2021/22 (36.9%), when SARS-CoV-2 predominated. A slight upward trend was observed in later seasons.

Similarly, the 15–29 age group showed limited variation, with a slight decreasing trend over time. More pronounced seasonal differences were observed in the 5–14 age group, with significantly lower proportions (adjusted *p* < 0.001) during 2020/21 (16.5%) and 2021/22 (19.6%).

The most substantial differences (adjusted *p* < 0.001) were observed in older age groups (30–64 and ≥65 years). During 2020/21 and 2021/22, the proportion of cases in the 30–64 age group was 1.4–3.3 times higher, and in the ≥65 age group 4.9–6.9 times higher compared to other seasons. This likely reflects the predominance of SARS-CoV-2 during this period, which disproportionately affected older individuals in terms of both infection risk and disease severity [26].

Comparison of the 2017/18–2019/20 seasons with the 2022/23–2024/25 seasons demonstrates an almost complete return to the pre-pandemic age distribution pattern, suggesting that the impact of SARS-CoV-2 circulation on overall ILI morbidity has progressively diminished.

Using weekly aggregated data on influenza-positive sentinel samples, the age distribution of laboratory-confirmed influenza cases was analyzed within the same age groups.

No significant differences in age distribution of influenza-positive cases were observed between pre- and post-2020/21 periods overall. However, certain seasonal patterns were identified.

In post-2020/21 seasons dominated by A(H3), a significant increase (adjusted *p* < 0.05) in the proportion of cases aged 15–29 years (1.4–1.8-fold) was observed. Conversely, during seasons characterized by joint dominance of A(H1)pdm09 and influenza B, the proportion of cases aged 15–29 years was significantly lower (adjusted *p* < 0.05).

No consistent patterns were identified in the 0–4 and 5–14 age groups. A more granular age stratification might reveal additional patterns; however, sentinel surveillance follows WHO-recommended age groupings [26] and more detailed stratification is currently unavailable. Further investigation is warranted.

In the ≥65 age group, a marked decline in the proportion of influenza-positive cases was observed during periods of active SARS-CoV-2 circulation (2019/20–2021/22). Potential explanations may include increased protective behaviors among older adults, targeted non-pharmaceutical interventions, prioritization of COVID-19 testing over influenza testing, higher vaccination coverage in this age group, or differential healthcare-seeking patterns during the pandemic. Similar trends have been reported in other settings, although causal mechanisms remain insufficiently clarified. Further analytical studies are required to determine the relative contribution of these factors.

### 3.3. Genetic Diversity of Influenza Viruses

During the 2017/18–2024/25 epidemic seasons, 48,711 respiratory specimens collected through sentinel surveillance were tested for influenza viruses by RT-PCR, of which 9043 were positive. Overall, 110 virus isolates were obtained in MDCK cells. Detailed numbers of specimens tested, viruses isolated, and sequences generated by epidemic season and virus subtype are provided in Supplementary Table S1. The resulting sequence dataset was used to characterize the genetic diversity and phylogenetic relationships of influenza A(H1N1)pdm09, A(H3N2), B/Victoria, and B/Yamagata viruses circulating in Kazakhstan.

#### 3.3.1. Influenza A (H1N1)pdm09

**Figure 2:**
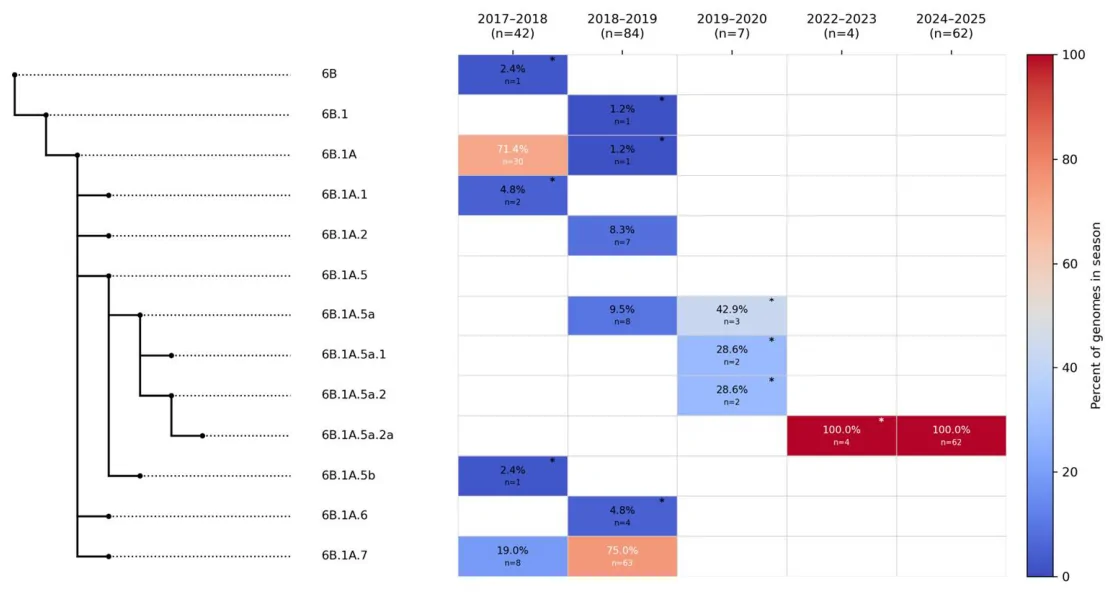
Influenza A(H1N1)pdm09 virus clade composition by epidemic season. Clades represented by fewer than five viruses in a given epidemic season are marked with an asterisk.

A total of 199 influenza A(H1N1)pdm09 viruses collected across the 2017/18–2024/25 epidemic seasons were genetically characterized. This period was marked by the diversification and spread of descendants of clade 6B in Kazakhstan (Figure 2).

**Figure 3:**
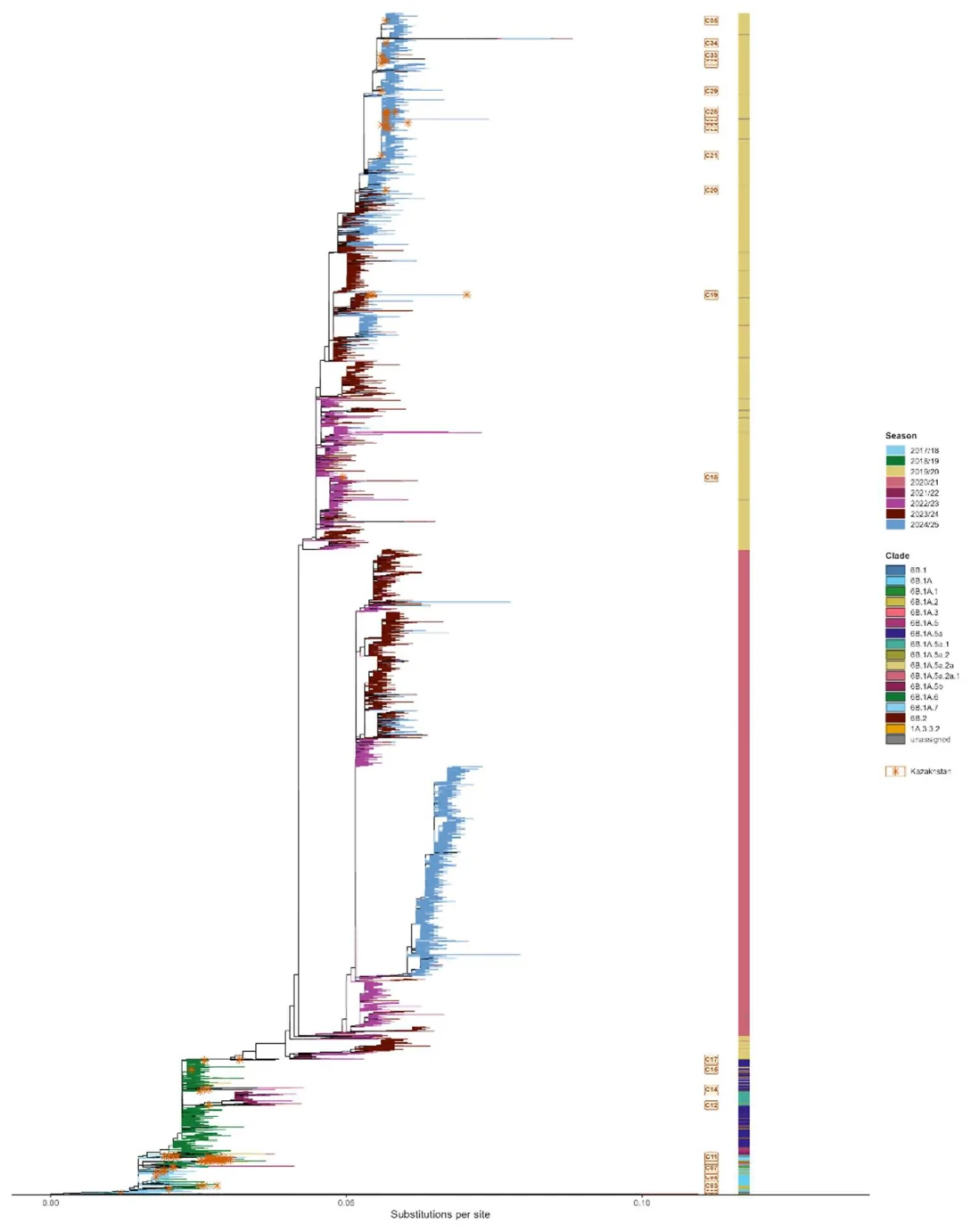
Maximum-likelihood phylogenetic tree of the HA gene of influenza A(H1N1)pdm09 viruses collected between 2017 and 2025. Viruses from Kazakhstan are indicated by orange asterisks. Phylogenetic clusters containing Kazakhstan viruses are labeled C01–C35. Subtrees corresponding to each phylogenetic cluster are provided in the Supplementary Figure S1.

Phylogenetic analysis was performed using all available HA sequences of influenza A(H1N1)pdm09 viruses collected during the 2017/18–2024/25 epidemic seasons. The closest background sequences sharing ≥99% nucleotide identity were selected from the global dataset, as described in the Materials and Methods. In total, 18,176 HA sequences were included in the final phylogenetic analysis (Figure 3).

In epidemic season 2017/18 the 6B.1A subclade predominated, accounting for 71.4% of viruses (Figure 2). This genetic subclade is defined by the HA1 amino acid substitutions S74R, S164T, which alters the glycosylation motif at residues 162–164, and I295V. Kazakhstan viruses belonging to subclade 6B.1A and collected during the 2017/18 epidemic season formed six phylogenetic clusters (C02, C04–C07, and C11). In the largest cluster, C11, four viruses showed high HA sequence similarity to strains from the Russian Federation, while viruses in cluster C05 grouped with viruses from Armenia. The remaining clusters included viruses of diverse geographic origin.

The second most common genetic clade during the 2017/18 season was 6B.1A.7, characterized by one substitution in the C-terminal region of HA1, K302T, and three substitutions in HA2: I404M, N496S, and E506D. Viruses belonging to clade 6B.1A.7 formed two phylogenetic clusters, C08 and C09, both of which also included viruses from Russia and Kyrgyzstan.

Clade 6B and subclades 6B.1A.1 and 6B.1A.5b accounted for 2.4%, 4.8%, and 2.4% of viruses, respectively.

All A(H1N1)pdm09 viruses from Kazakhstan collected during the 2017/18 epidemic season were antigenically indistinguishable from the egg-propagated vaccine virus A/Michigan/45/2015, being recognized at titers within twofold of the homologous antiserum titer (See Supplementary Table S2). No antigenic differences were observed between clade 6B.1A and its descendant genetic subclades 6B.1A.1 and 6B.1A.7.

Subclade 6B.1A.7 became dominant during the 2018/19 epidemic season, accounting for 75% of the characterized viruses. Kazakhstan viruses belonging to this subclade formed three phylogenetic clusters (C08–C10) in the global phylogenetic tree. Viruses in clusters C08 and C09 were genetically closely related to Kazakhstan viruses collected during the preceding 2017/18 season, as well as to viruses from Kyrgyzstan, Russia, and several European countries, including Estonia and Hungary.

The second most common subclade in this season was 6B.1A.5a, which accounted for 9.5% and was defined by the HA1 substitutions N260D, N129D, and T185I. This subclade subsequently expanded to 42.9% during the 2019/20 epidemic season in Kazakhstan and further diverged into 6B.1A.5a.1 and 6B.1A.5a.2. The signature amino acid substitutions of 6B.1A.5a.1 were D187A and Q189E, whereas 6B.1A.5a.2 was characterized by N156K, L161I, and V250A. A representative of clade 6B.1A.5a.1, A/Guangdong-Maonan/SWL1536/2019, was recommended as an egg-based influenza vaccine strain for the 2020–2021 Northern Hemisphere influenza season.

**Figure 4:**
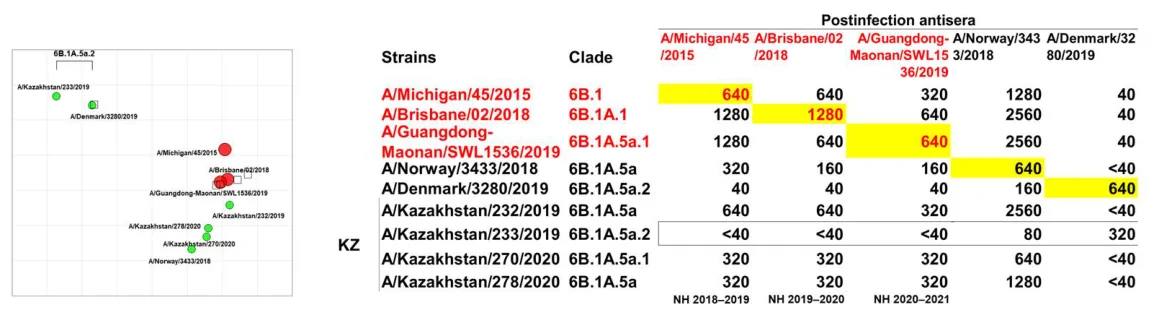
Antigenic analysis of influenza A(H1N1pdm09) viruses from Kazakhstan from 2019/20 epidemic season; antigenic map (**left**) and corresponding HI results (**right**). Antigenic distances are expressed in antigenic units (AU), with one grid unit corresponding to a 2-fold difference in HI titer. Box indicates titers for 6B.1A.5a.2 from Kazakhstan. Raw antigenic data were kindly provided by Worldwide Influenza Centre at Francis Crick Institute (UK). Vaccine strains are shown in red, and homologous titers are highlighted in yellow.

Substantial changes in the antigenic properties of influenza A(H1N1)pdm09 viruses belonging to subclade 6B.1A.5a.2 were observed. Antisera raised against egg-propagated A/Guangdong-Maonan/SWL1536/2019 (subclade 6B.1A.5a.1) and the previous vaccine strains A/Brisbane/02/2018 (clade 6B.1A.1) and A/Michigan/45/2015 (clade 6B.1) recognized A/Kazakhstan/233/2019 (clade 6B.1A.5a.2) at titers that were 16- to 32-fold lower than the corresponding homologous titers (see Figure 4). In contrast, antiserum raised against A/Denmark/3280/2019 (clade 6B.1A.5a.2) recognized A/Kazakhstan/233/2019 at a titer within 2-fold of the homologous titer.

No genetic data for influenza A(H1N1)pdm09 viruses were obtained in Kazakhstan during the first two years of the COVID-19 pandemic, corresponding to the 2020/21 and 2021/22 epidemic seasons. Genetic data from the 2022/23 epidemic season were very limited; however, descendant lineage 6B.1A.5a.2a was found to predominate among influenza A(H1N1)pdm09 viruses. The transition from clade 6B.1A.5a.2 to 6B.1A.5a.2a was not accompanied by detectable antigenic changes. In the HI assay, A/Almaty Region/12162/2022 (6B.1A.5a.2a) was recognized at the homologous titer by antisera raised against A/Denmark/3280/2019 (clade 6B.1A.5a.2) and the egg-propagated vaccine strain A/Victoria/2570/2019 (6B.1A.5a.2).

During the 2024/25 epidemic season, descendants of subclade 6B.1A.5a.2a, namely genetic lineages C.1.9 and C.1.9.3, predominated in Kazakhstan, accounting for 27.4% and 72.6% of viruses, respectively. Subclade 6B.1A.5a.2a viruses from Kazakhstan formed 17 phylogenetic clusters (C19–C35; see Supplementary Figure S1). Most clusters contained viruses of diverse geographic origin, whereas cluster C28 consisted predominantly of viruses from Russia.

**Figure 5:**
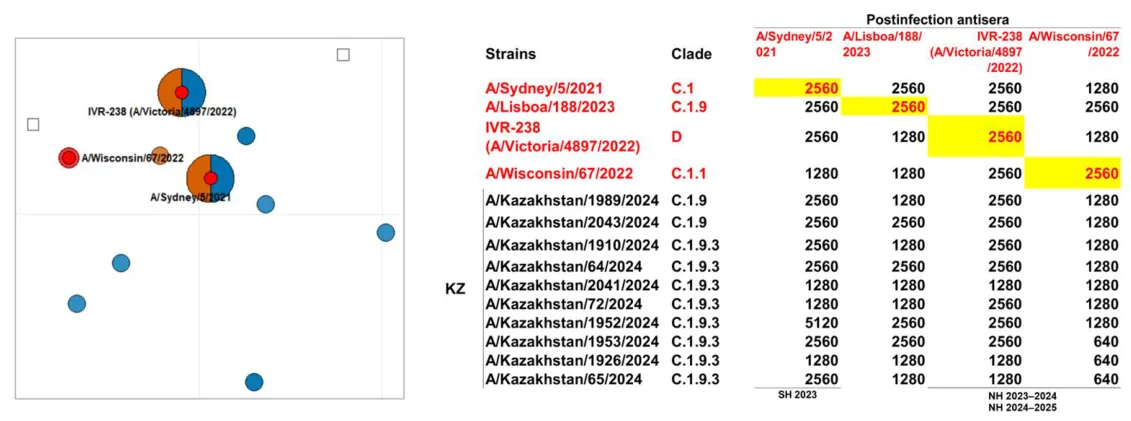
Antigenic analysis of influenza A(H1N1pdm09) viruses from Kazakhstan from 2024/25 epidemic season; antigenic map (**left**) and corresponding HI results (**right**). Antigenic distances are expressed in antigenic units (AU), with one grid unit corresponding to a 2-fold difference in HI titer. Vaccine strains are shown in red, and homologous titers are highlighted in yellow. Clade C.1.9 viruses are shown in brown, clade C.1.9.3 in dark blue. Raw antigenic data were kindly provided by Worldwide Influenza Centre at Francis Crick Institute (UK).

Lineage C.1.9 was characterized by the substitutions T120A and K169Q, while sublineage C.1.9.3 additionally possessed S83P and I510T. No antigenic differences between 6B.1A.5a.2a (C.1) and its descendants C.1.9 and C.1.9.3 were observed in the HI assay. Post-infection ferret antisera raised against cell-propagated A/Sydney/5/2021 (6B.1A.5a.2a) recognized both C.1.9 and C.1.9.3 lineage isolates from Kazakhstan at homologous titers (Figure 5).

Screening of the analyzed A(H1N1)pdm09 virus sequences collected throughout the 2017/18–2024/25 epidemic seasons did not identify any established molecular markers previously associated with antiviral resistance.

#### 3.3.2. Influenza A (H3N2)

**Figure 6:**
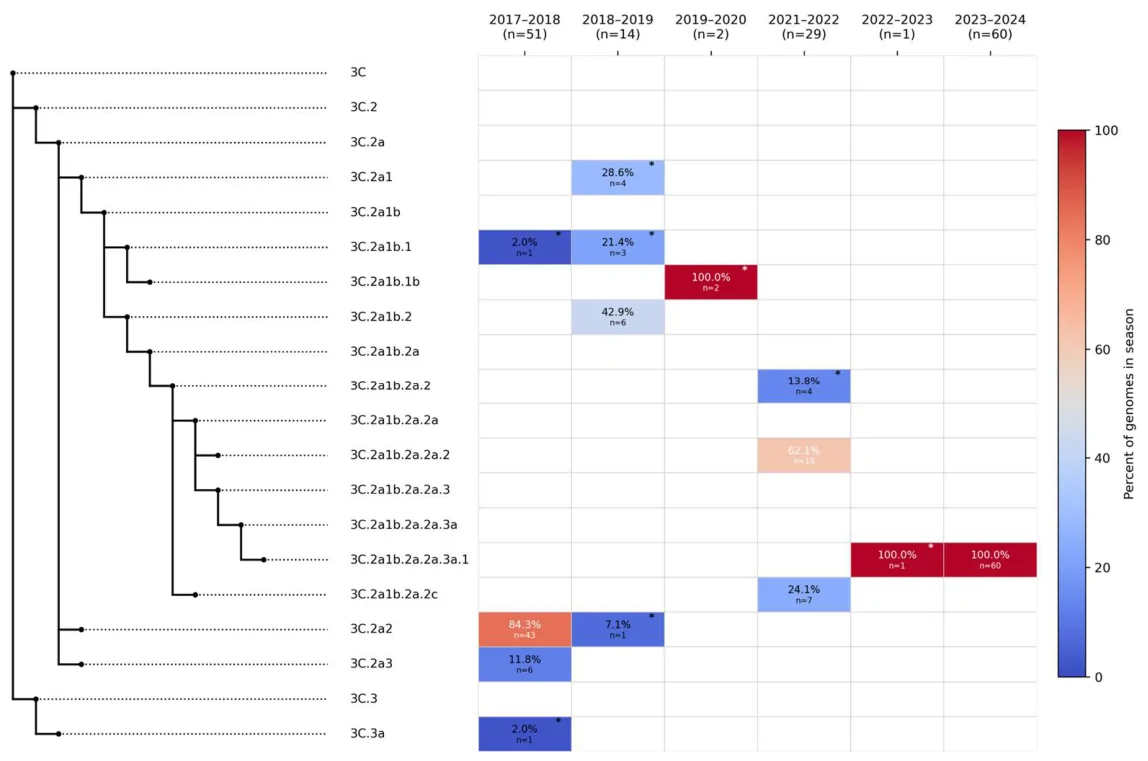
Influenza A(H3N2) virus clade composition by epidemic season. Clades represented by fewer than five viruses in a given epidemic season are marked with an asterisk.

A total of 159 influenza A(H3N2) viruses collected across the 2017/18–2024/25 epidemic seasons were genetically characterized. This period was marked by the diversification and spread of descendants of clade 3C in Kazakhstan (Figure 6).

**Figure 7:**
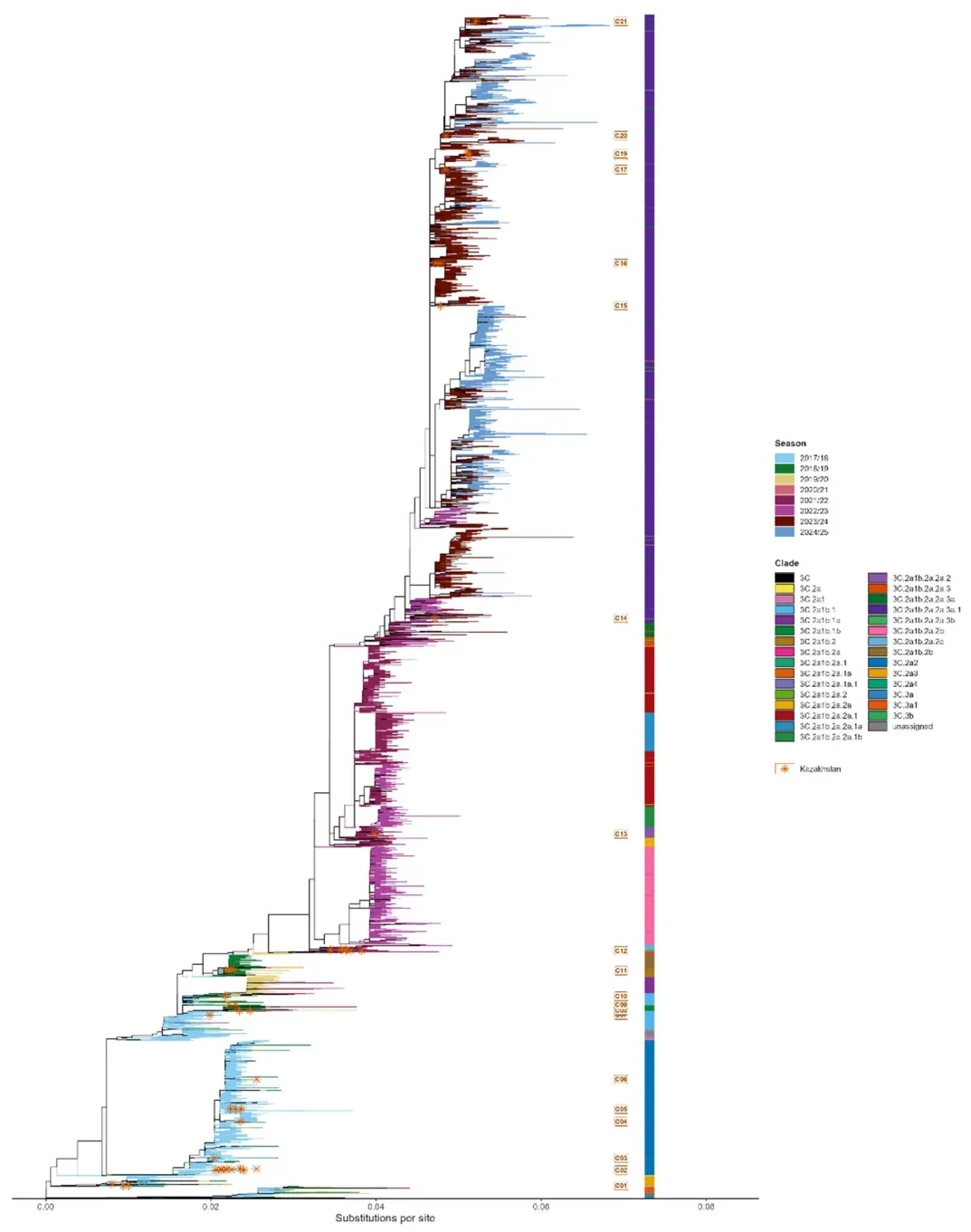
Maximum-likelihood phylogenetic tree of the HA gene of influenza A(H3N2) viruses collected between 2017 and 2025. Viruses from Kazakhstan are indicated by orange asterisks. Phylogenetic clusters containing Kazakhstan viruses are labeled C01–C21. Subtrees corresponding to each phylogenetic cluster are provided in the Supplementary Figure S2.

Phylogenetic analysis was performed using all available HA sequences of influenza A(H3N2) viruses collected during the 2017/18–2024/25 epidemic seasons. The closest background sequences sharing ≥99% nucleotide identity were selected from the global dataset, as described in the Materials and Methods. In total, 12,831 HA sequences were included in the final phylogenetic analysis (Figure 7).

During the 2017/18 epidemic season, descendants of subclade 3C.2a predominated, accounting for 98% of viruses: 3C.2a.2, 84.3%; 3C.2a.3, 11.0%; and 3C.2a1b.1, 2.0%. The remaining 2% belonged to clade 3C.3a (Figure 5). Kazakhstan viruses collected during the 2017/18 epidemic season formed six phylogenetic clusters (C01–C05 and C07), together with viruses of diverse geographic origin (see Supplementary Figure S2). Viruses from Kazakhstan in cluster C07 were genetically closely related to viruses from Russia.

Clade 3C.2a was characterized by the following HA1 substitutions: L3I, N128T, resulting in the gain of a potential glycosylation site; N144S, resulting in the loss of a potential glycosylation site; N145S, F159Y, K160T, resulting in the gain of a potential glycosylation site; V186G, P198S, F219S, N225D, and Q311H. Subclade 3C.2a.2 was defined by the HA1 substitutions T131K, R142K, and R261Q. Clade 3C.3a possessed several characteristic HA1 substitutions: T128A, resulting in the loss of a potential glycosylation site; A138S, R142G, N145S, F159S, V186G, P198S, F219S, and N225D. Clade 3C.3a was not detected in Kazakhstan after the 2017/18 epidemic season. All subsequent epidemic seasons in Kazakhstan were marked by the circulation of descendants of clade 3C.2a.

During the 2018/19 epidemic season, the proportion of influenza A(H3N2) viruses belonging to clade 3C.2a.2 in Kazakhstan decreased from 84.3% to 7.1%. No single predominant clade was identified, with clades 3C.2a1, 3C.2a1b.1, and 3C.2a1b.2 circulating at frequencies of 28.6%, 21.4%, and 42.8%, respectively. Subclade 3C.2a1 was defined by the HA1 substitutions N121K and N171K, together with the HA2 substitutions I77V, G155E, and G160E. Additional HA1 substitutions K92R and H311Q defined the large subclade 3C.2a1b. Subclade 3C.2a1b.1 was defined by T128A and T135K, both resulting in the loss of potential glycosylation sites, with most viruses also possessing A138S and F193S in HA1.

During the 2019/20 epidemic season, only two influenza A(H3N2) viruses were genetically characterized. Both belonged to subclade 3C.2a1b.1b, formerly designated 3C.2a1b + T135K-B, which additionally possessed the HA1 substitution S137F. A/Kansas/14/2017-like viruses, belonging to clade 3C.3a, were recommended by the WHO as the A(H3N2) vaccine component for the 2019/20 Northern Hemisphere influenza season. Antisera raised against egg-propagated NYMC X-327 A/Kansas/14/2017, subclade 3C.3a, recognized influenza virus strains from Kazakhstan, clade 3C.2a1b.1, at titers that were 8- to 16-fold lower than the corresponding homologous titers (see Supplementary Table S2).

During the 2020/21 epidemic season, no influenza A(H3N2) viruses were genetically characterized in Kazakhstan. The 2021/22 epidemic season was dominated by clade 3C.2a1b.2a.2a.2, which accounted for 62.1% of viruses, followed by 3C.2a1b.2a.2c and 3C.2a1b.2a.2, accounting for 24.1% and 13.8%, respectively.

In the following epidemic seasons, 2022/23 and 2023/24, influenza A(H3N2) viruses in Kazakhstan were dominated by clade 3C.2a1b.2a.2a.3a.1. All A(H3N2) virus HA sequences from Kazakhstan collected during the 2023/24 epidemic season clustered within clade 2a.3a.1, subclade J.2, except for one sequence, which clustered within subclade J.

Throughout the 2017/18-2024/25 epidemic seasons, none of the analyzed A(H3N2) virus sequences harbored established molecular markers previously associated with antiviral resistance.

#### 3.3.3. Influenza B/Victoria

**Figure 8:**
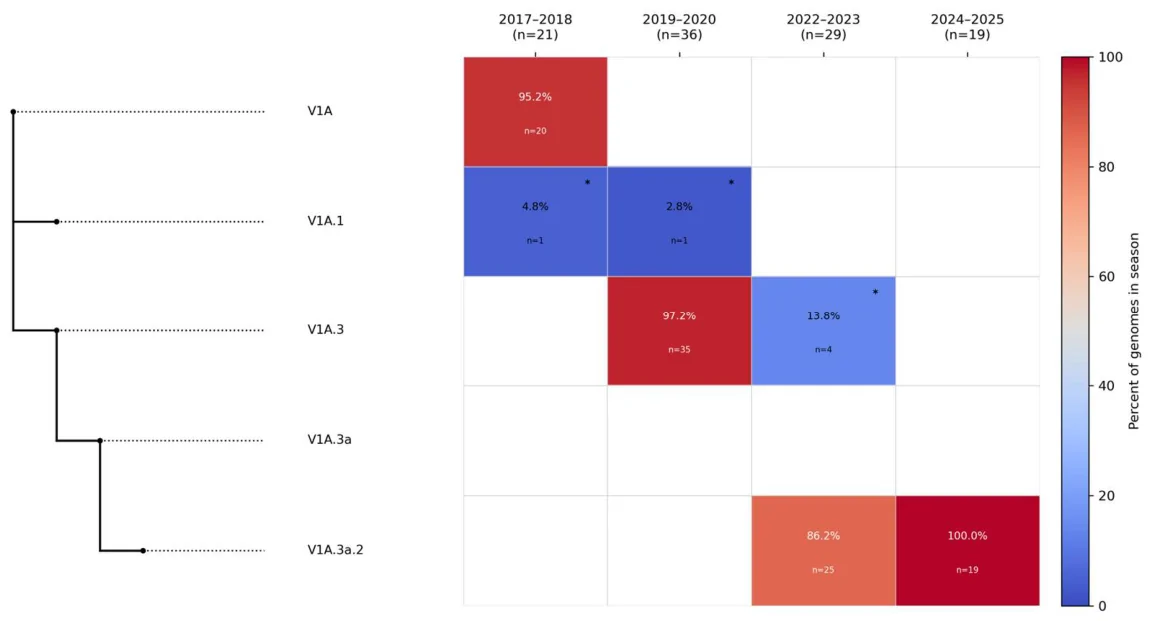
Influenza B(Victoria) virus clade composition by epidemic season. Clades represented by fewer than five viruses in a given epidemic season are marked with an asterisk.

A total of 105 influenza B/Victoria viruses collected across the 2017/18–2024/25 epidemic seasons were genetically characterized. The genetic structure of influenza B/Victoria viruses changed substantially over the analyzed seasons (Figure 8). During the 2017/18 epidemic season, V1A remained predominant, accounting for 95% of genomes, while the descendant clade V1A.1 was detected at low frequency, representing less than 5%. No B/Victoria viruses were sequenced in Kazakhstan in 2018/2019 epidemic season. By the 2019/20 season, earlier V1A/V1A.1 viruses had been nearly completely replaced by clade V1A.3, which accounted for 97.2% of analyzed genomes. In 2022/23, further diversification was observed, with the emergence and predominance of V1A.3a.2, which represented 86.2% of genomes, while V1A.3 still accounted for 13.8%. Thereafter, V1A.3a.2 became the dominant lineage, increasing to 100% in 2024/25. Overall, the data indicate successive clade replacement from V1A to V1A.3 and subsequently to V1A.3a.2, which became fixed among the analyzed B/Victoria viruses in recent seasons.

Phylogenetic analysis was performed using all available HA sequences of influenza B/Victoria viruses collected during the 2017/18–2024/25 epidemic seasons. The closest background sequences sharing ≥99% nucleotide identity were selected from the global dataset, as described in the Materials and Methods. In total, 13,297 HA sequences were included in the final phylogenetic analysis, and 16 phylogenetic clusters were identified.

**Figure 9:**
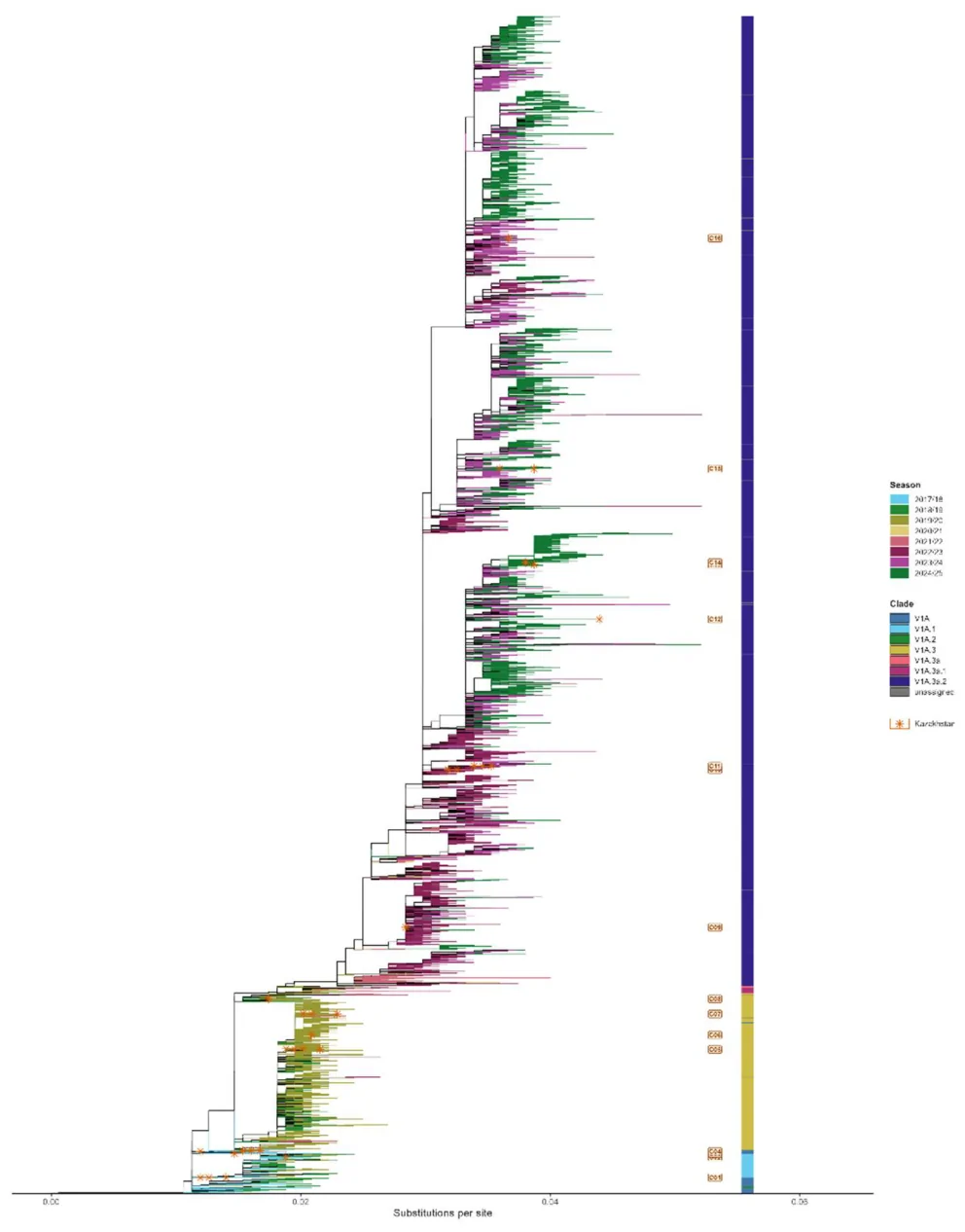
Maximum-likelihood phylogenetic tree of the HA gene of influenza B/Victoria viruses collected between 2017 and 2025. Viruses from Kazakhstan are indicated by orange asterisks. Phylogenetic clusters containing Kazakhstan viruses are labeled C01–C16. Subtrees corresponding to each phylogenetic cluster are provided in the Supplementary Figure S3.

During the 2017/18 epidemic season, influenza B/Victoria viruses from Kazakhstan formed four phylogenetic clusters (C01–C04) (Figure 9; see Supplementary Figure S3 for details). All clusters belonged to clade V1A, except C02, which belonged to V1A.1. Kazakhstan V1A viruses (B/Brisbane/60/2008-like) collected during the 2017/18 season clustered predominantly with viruses from Asia, including China and Japan. In contrast, B/Kazakhstan/346/2018, which belonged to V1A.1 (also known as the Δ2 group or B/Colorado/06/2017-like viruses), grouped with European viruses, including strains from Poland.

During the 2019/20 epidemic season, B/Victoria viruses belonging to clade V1A.3 (also known as the Δ3 group or B/Washington/02/2019-like viruses) formed three phylogenetic clusters (C05–C07). Cluster C05 included viruses from Russia, the USA, Canada, and several European countries. In cluster C06, B/Kazakhstan/9379/2019 grouped with a virus from Germany, whereas Kazakhstan viruses in cluster C07 were phylogenetically closely related to viruses from different regions of Russia.

**Figure 10:**
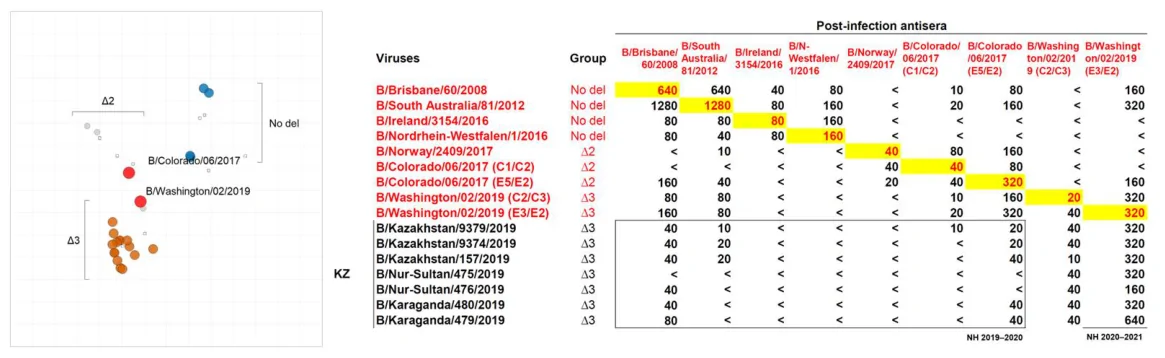
Antigenic analysis of influenza A(H1N1)pdm09 viruses from Kazakhstan collected during the 2019/20 epidemic season: antigenic map (**left**) and corresponding HI results (**right**). Vaccine strains are shown in red, homologous titers are highlighted in yellow, B/Brisbane/60/2008-like viruses lacking deletion are shown in blue, viruses from Kazakhstan are shown in brown, other viruses are shown in grey. Antigenic distances are expressed in antigenic units (AU), with one grid unit corresponding to a 2-fold difference in HI titer. Raw antigenic data were kindly provided by the Worldwide Influenza Centre at the Francis Crick Institute, UK.

The Δ2 and Δ3 B/Victoria viruses carry deletions at HA1 positions 162–163 and 162–164, respectively, within the 160-loop, resulting in substantial antigenic changes as demonstrated by HI titers. Kazakhstan Δ3 viruses showed 8- to 128-fold reductions in HI titers relative to the homologous titers of post-infection ferret antisera raised against egg-propagated B/Brisbane/60/2008-like viruses lacking a deletion in the HA1 160-loop, and 4- to 8-fold reductions relative to Δ2 (B/Colorado/06/2017-like) viruses (Figure 10). Consistent with these findings, the antigenic map showed clear separation among B/Brisbane/60/2008-like viruses (no deletion), B/Colorado/06/2017-like viruses (Δ2), and B/Washington/02/2019-like viruses (Δ3).

During the 2022/23 epidemic season, B/Victoria viruses belonging to clade V1A.3 (also known as the Δ3 group or B/Washington/02/2019-like viruses) formed a single phylogenetic cluster (C08) closely related to European viruses, including strains from Russia, and to viruses from the Eastern Mediterranean Region collected during the 2018/19 and 2019/20 epidemic seasons. In contrast, the vast majority of Kazakhstan viruses belonged to clade V1A.3a.2 and formed three phylogenetic clusters (C09–C11). Cluster C09 included viruses of diverse geographic origin, whereas clusters C10 and C11 predominantly contained viruses from Eastern Europe, including Latvia, Ukraine, and Russia, and Central Asia, including Kyrgyzstan. In addition to the Δ162–164 deletion, clade V1A.3a.2 viruses carried the HA1 substitutions A127T and P144L. No antigenic differences were observed between Kazakhstan V1A.3a.2 viruses and B/Austria/1359417/2021-like viruses recommended for use in Northern Hemisphere influenza vaccines for the 2022/23 season.

No influenza B viruses were genetically characterized during the 2023/24 epidemic season. In 2024/25, B/Victoria viruses belonging to clade V1A.3a.2 formed five phylogenetic clusters (C12–C16). Kazakhstan viruses in cluster C16 grouped clearly with viruses from the United Arab Emirates, whereas the remaining clusters contained viruses from diverse geographic locations. Clade V1A.3a.2 viruses also acquired the HA1 substitution E183K during the 2024/25 epidemic season.

Genotypic analysis of B/Victoria virus sequences collected during the 2017/18-2024/25 epidemic seasons revealed no established molecular substitutions previously associated with antiviral resistance.

#### 3.3.4. Influenza B/Yamagata

The genetic data of influenza B/Yamagata viruses from Kazakhstan exists only for 2017/18 epidemic season—16 viruses were genetically characterized. No B/Yamagata viruses were detected in Kazakhstan since that time. All viruses belong to genetic clade 3 and were antigenically similar to vaccine strain B/Phuket/3073/2013 (see Supplementary Table S2).

**Figure 11:**
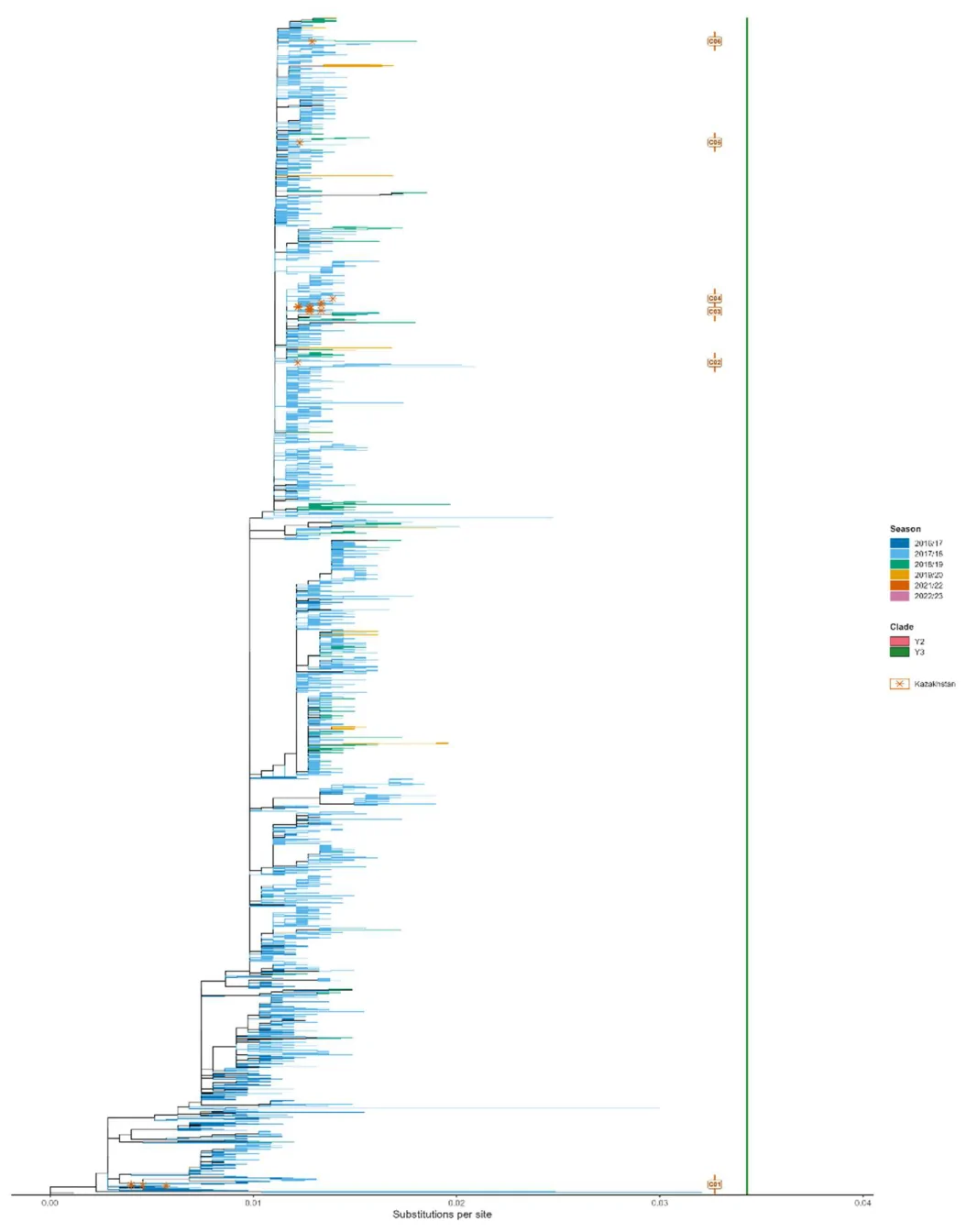
Maximum-likelihood phylogenetic tree of the HA gene of influenza B/Yamagata viruses collected between 2017 and 2020. Viruses from Kazakhstan are indicated by orange asterisks. Phylogenetic clusters containing Kazakhstan viruses are labeled C01–C06. Subtrees corresponding to each phylogenetic cluster are provided in the Supplementary Figure S4.

Phylogenetic analysis was performed using all available HA sequences of influenza B/Yamagata viruses collected during the 2017/18–2019/20 epidemic seasons. The closest background sequences sharing ≥99% nucleotide identity were selected from the global dataset, as described in the Materials and Methods. In total, 2360 HA sequences were included in the final phylogenetic analysis, and six phylogenetic clusters were identified (Figure 11).

Most of the viruses from Kazakhstan in epidemic season 2017/18 belonged to clusters C03 and C04 (see Supplementary Figure S4) and were genetically close to viruses from Russia and Kyrgyzstan.

Genotypic screening of the 16 available B/Yamagata virus sequences from the 2017/18 epidemic season did not identify any established molecular markers previously associated with antiviral resistance.

## 4. Discussion

This study provides a multi-season overview of influenza circulation, genetic diversification, and antigenic properties in Kazakhstan across the pre-pandemic, pandemic, and post-pandemic periods. Overall, Kazakhstan followed the broad evolutionary trends observed across the WHO European Region, but often differed in the timing of epidemics and in the dominant virus type or subtype.

During 2017/18–2019/20, A(H1N1)pdm09, A(H3N2), and influenza B viruses co-circulated or circulated sequentially. In 2017/18, Kazakhstan differed from much of Europe, where B/Yamagata predominated strongly [27], whereas in 2018/19 the epidemiological pattern was more similar to that of the WHO European Region, with A(H1N1)pdm09 predominating over A(H3N2) and limited influenza B activity [28]. The 2019/20 season was characterized by early influenza B activity followed by co-circulation of A(H1N1)pdm09 and A(H3N2), broadly consistent with the European pattern before influenza activity was disrupted by the emergence of SARS-CoV-2 [29].

Influenza was almost completely absent in Kazakhstan during 2020/21 despite restoration of testing capacity. This was consistent with the unprecedented reduction in influenza activity observed globally during the COVID-19 pandemic; in the WHO European Region, sentinel influenza-positive detections decreased by 99.8% compared with previous seasons [23,30]. Influenza re-emerged in 2021/22, predominantly as A(H3N2), paralleling the wider regional return of clade 3C.2a1b.2a.2 viruses [31,32,33].

More pronounced differences from EU/EEA countries were observed after the pandemic. In 2022/23, Kazakhstan experienced early influenza B predominance followed by A(H1N1)pdm09, whereas A(H3N2) initially predominated in much of Europe [34]. In 2023/24, Kazakhstan was A(H3N2)-dominated, while A(H1N1)pdm09 predominated in EU/EEA surveillance [35]. Nevertheless, Kazakhstan A(H3N2) viruses largely belonged to clade 2a.3a.1/J.2, consistent with the broader global evolution of this subtype [36,37].

The genetic data confirm the rapid evolutionary turnover characteristic of A(H3N2). Kazakhstan viruses shifted from 3C.2a descendants in 2017/18 to multiple 3C.2a1b-related subclades in 2018/19, then to 3C.2a1b.2a.2-related viruses and finally to 2a.3a.1/J.2 viruses in 2023/24 [38]. In contrast, A(H1N1)pdm09 showed a more stepwise pattern, with succession of 6B-derived lineages and later predominance of 6B.1A.5a.2a descendants.

The antigenic changes observed in Kazakhstan were broadly consistent with contemporaneous trends in Europe and Russia. For A(H1N1)pdm09, the emergence of 6B.1A.5a.2 was associated with reduced recognition by antisera to earlier vaccine strains, whereas subsequent diversification into 6B.1A.5a.2a and C.1 descendants produced little detectable antigenic change, in agreement with European virus characterization data [30]. Kazakhstan A(H3N2) viruses from 2019/20 also showed reduced reactivity to A/Kansas/14/2017 antiserum, paralleling European and Russian reports of antigenic mismatch among 3C.2a1b viruses, including 137F variants [30,39]. Similarly, the marked antigenic separation of B/Victoria no-deletion, Δ2, and Δ3 viruses was consistent with the broader regional emergence of antigenically distinct V1A.3/Δ3 viruses. Together, these findings emphasize the value of combining genetic and antigenic surveillance, as phylogenetic diversification does not necessarily correspond directly to antigenic drift.

Influenza B evolution in Kazakhstan also reflected global post-pandemic trends. B/Victoria viruses showed progressive replacement of V1A by V1A.3 and subsequently V1A.3a.2, while naturally circulating B/Yamagata-lineage viruses disappeared after 2020 [40,41].

Differences in subtype dominance between Kazakhstan and European countries may partly reflect variation in the timing and sources of virus introductions. Kazakhstan’s geographic position between Europe and Asia and its epidemiological links with Russia and other Central Asian countries make repeated introductions from several regional sources plausible. Global phylogeographic studies have shown that seasonal influenza viruses are maintained through recurrent inter-regional migration rather than uninterrupted local persistence [42]. Previous studies have also identified Kazakhstan A(H3N2) viruses genetically related to viruses circulating in several regions of Russia, as well as in North America, China, Croatia, and other countries [15,41]. Similarly, clade 6B.1A.5a.2a A(H1N1)pdm09 viruses predominated in both Kazakhstan and Russia during 2022/23 [43,44]. These findings are consistent with regional circulation of closely related viruses, although they do not establish specific transmission routes or sources.

Regional heterogeneity within Central Asia may also contribute to the differences observed between Kazakhstan and EU/EEA countries. Surveillance data have shown that different influenza types and subtypes may predominate simultaneously in neighboring Central Asian countries [45]. Thus, the epidemiological patterns observed in Kazakhstan likely reflect both broader Eurasian circulation and local or regional transmission dynamics.

Human mobility provides a plausible mechanism for this connectivity. Global phylogeographic analyses have demonstrated recurrent geographic migration of seasonal influenza A viruses, with no single consistent direction of global spread [40]. Air travel has been identified as an important driver of long-distance A(H3N2) migration, while other mobility processes contribute at regional and local scales [46]. Seasonal variation in migration may also influence the probability that newly introduced lineages become successfully established [47], while regional seasonality, population size, and transmission potential may further shape lineage persistence and dominance [48].

However, the present study was designed for virological surveillance rather than formal phylogeographic or phylodynamic reconstruction. The sampling density is insufficient to resolve individual transmission chains or reliably distinguish local persistence from repeated introductions; therefore, phylogenetic relationships should be interpreted as evidence of regional connectivity rather than definitive evidence of geographic source or transmission direction.

Overall, Kazakhstan was integrated into the broader evolutionary dynamics of seasonal influenza across Eurasia, but frequently exhibited distinct timing and subtype dominance. These findings underline the importance of maintaining integrated epidemiological, antigenic, and genomic surveillance in Kazakhstan. Continued timely sharing of representative sequence and antigenic data through GISRS is important for regional risk assessment, vaccine strain selection, and understanding influenza evolution across the WHO European Region.

## 5. Conclusions

This study provides an integrated multi-season analysis of the antigenic, molecular genetic, phylogenetic, and epidemiological evolution of influenza viruses circulating in Kazakhstan over eight consecutive epidemic seasons (2017–2025). By integrating epidemiological surveillance, antigenic characterization, sequencing, and phylogenetic analysis, we characterized the circulation dynamics of A(H1N1)pdm09, A(H3N2), and influenza B viruses across the pre-pandemic, pandemic, and post-pandemic periods.

The results demonstrated substantial changes in influenza epidemiology following the COVID-19 pandemic, including shifts in epidemic timing, alternating dominance of influenza virus types and subtypes, and changes in genetic diversity across epidemic seasons. Molecular characterization showed that influenza viruses circulating in Kazakhstan belonged to globally recognized genetic clades and accumulated characteristic amino acid substitutions associated with ongoing antigenic evolution.

Continuous sequencing and antigenic monitoring are essential for the early detection of emerging variants, assessment of vaccine match, monitoring of antiviral resistance markers, and timely public health decision-making.

Overall, the findings presented in this study strengthen the evidence base for influenza surveillance in Kazakhstan and contribute to the Global Influenza Surveillance and Response System (GISRS) by improving knowledge of regional influenza virus evolution and supporting national and international influenza prevention and control strategies.

## 6. Limitations

Several limitations should be considered when interpreting the findings of this surveillance study. These mainly relate to variation in sampling intensity across epidemic seasons, incomplete genomic coverage during the COVID-19 pandemic period, and the limited ability of routine surveillance data to resolve detailed transmission chains or distinguish local persistence from repeated virus introductions. This study was designed as a national influenza surveillance study rather than a transmission-chain or phylodynamic investigation; the number and temporal density of sequenced viruses are insufficient to robustly reconstruct individual introductions, local persistence, or transmission chains. Accordingly, our phylogenetic results are interpreted primarily in terms of genetic diversity and clade composition rather than detailed transmission dynamics.

When moving from the 2011 to the 2014 WHO case definitions, the changes in the ILI and SARI definitions have different implications for the analysis. For ILI, the only change was the maximum interval between symptom onset and presentation, which increased from 7 to 10 days. Other ILI criteria remained unchanged, including a temperature of ≥38 °C and cough. In the context of modern RT-PCR testing, the additional 3-day interval is unlikely to substantially affect the detection of respiratory viruses. Therefore, the main effect of the change in the ILI definition is an increase in the number of patients meeting the ILI case definition due to the less restrictive time criterion (10 days instead of 7 days). In contrast, the changes in the SARI case definition included substantial modifications to the criteria related to disease severity. Therefore, the most of SARI indicators based on the 2011 and 2014 case definitions are not directly comparable. Furthermore, the timing of the introduction of the new case definitions complicated the analysis, as they were implemented between the pre- and post-COVID-19 periods. This substantially limits the comparability of indicators between these two periods. Thus, this change completely prevented the use of SARI data, but it did allow for limited use of ILI data.

An important limitation of this study is that antiviral resistance was assessed exclusively at the genotypic level. Therefore, our findings should not be interpreted as confirmation of phenotypic antiviral resistance or susceptibility.

## Figures and Tables

**Table 1:** Age distribution among all ILI cases, 2017/18–2024/25.

Season	Age Groups				
	0–4	5–14	15–29	30–64	65+
2017/18 A(H1)pdm09 + A(H3) + B	53.3% (52.4–54.2%)	29.5% (28.7–30.3%)	10.0% (9.5–10.5%)	4.0% (3.6–4.3%)	3.2% (2.9–3.5%)
2018/19 A(H1)pdm09 + A(H3) → B	36.3% (35.3–37.3%)	32.6% (31.6–33.5%)	16.9% (16.1–17.7%)	6.3% (5.8–6.8%)	8.0% (7.5–8.6%)
2019/20 B → A(H1)pdm09 + A(H3)	40.9% (39.8–42.0%)	26.8% (25.8–27.8%)	17.6% (16.8–18.5%)	6.4% (5.9–7.0%)	8.2% (7.6–8.8%)
2020/21 No influenza	38.7% (37.7–39.7%)	16.5% (15.7–17.2%)	16.1% (15.4–16.9%)	13.0% (12.3–13.7%)	15.7% (15.0–16.4%)
2021/22 A(H3)	36.9% (36.0–37.8%)	19.6% (18.8–20.3%)	16.4% (15.7–17.1%)	8.5% (8.0–9.0%)	18.6% (17.9–19.3%)
2022/23 B → A(H1)pdm09	44.5% (43.8–45.1%)	27.9% (27.3–28.5%)	13.7% (13.2–14.1%)	5.8% (5.5–6.1%)	8.1% (7.8–8.5%)
2023/24 A(H3)	47.0% (46.5–47.6%)	29.9% (29.4–30.3%)	13.1% (12.7–13.4%)	5.3% (5.1–5.5%)	4.8% (4.5–5.0%)
2024/25 A(H1)pdm09 → B	50.5% (50.0–51.0%)	27.0% (26.5–27.4%)	13.7% (13.4–14.1%)	6.1% (5.9–6.4%)	2.7% (2.5–2.9%)

Source: Local electronic influenza surveillance system “Monitoring of ARVI/Influenza/Pneumonia”https://ilisari.kz/ (accessed on 25 July 2026).

**Table 2:** Age distribution among influenza-positive ILI cases, 2017/18–2024/25.

Season	Age Groups				
	0–4	5–14	15–29	30–64	65+
2017/18 A(H1)pdm09 + A(H3) + B	11.0% (7.5–14.7%)	20.0% (15.5–24.7%)	21.1% (16.4–25.8%)	26.1% (20.9–31.0%)	21.8% (17.0–26.6%)
2018/19 A(H1)pdm09 + A(H3) → B	18.7% (13.7–23.6%)	28.0% (22.2–33.7%)	13.0% (8.8–17.4%)	20.0% (14.8–25.0%)	20.2% (15.2–25.5%)
2019/20 B → A(H1)pdm09 + A(H3)	18.5% (13.8–23.4%)	29.3% (23.6–34.9%)	21.5% (16.3–26.4%)	18.1% (13.4–22.9%)	12.7% (8.5–16.7%)
2020/21	No influenza positives				
2021/22 A(H3)	13.8% (7.9–19.8%)	32.1% (24.3–40.3%)	30.2% (22.1–37.9%)	21.1% (13.7–27.8%)	2.8% (0.3–5.9%)
2022/23 B → A(H1)pdm09	16.3% (12.3–20.2%)	24.9% (20.2–29.4%)	18.8% (14.7–23.0%)	24.2% (19.6–28.7%)	15.8% (12.1–19.8%)
2023/24 A(H3)	14.5% (10.6–18.1%)	19.7% (15.5–24.0%)	27.1% (22.2–31.7%)	17.0% (13.0–21.1%)	21.8% (17.4–26.3%)
2024/25 A(H1)pdm09→B	17.7% (14.6–20.9%)	28.5% (24.7–32.1%)	16.6% (13.6–19.7%)	23.8% (20.4–27.4%)	13.4% (10.7–16.3%)

Source: Local electronic influenza surveillance system “Monitoring of ARVI/Influenza/Pneumonia” https://ilisari.kz/ Whether it should be https://ilisari.kz/, same as below).

## Data Availability

The nucleotide sequences generated in this study have been deposited in the GISAID EpiFlu database: EPI_SET_260804hw (DOI: https://doi.org/10.55876/gis8.260804hw).

## Supplementary Materials

The following supporting information can be downloaded at https://www.mdpi.com/article/10.3390/biology15181640/s1: Supplementary Table S1. Laboratory results from sentinel surveillance for influenza-like illness (ILI) and severe acute respiratory infection (SARI) in Kazakhstan, by virus subtype, from week 40 to week 20 of the 2017/18–2024/25 epidemic seasons; Supplementary Table S2. Hemagglutination inhibition (HI) data for antigenic characterization of influenza viruses from Kazakhstan; Supplementary Figure S1. Phylogenetic subtrees and geographic composition of clusters containing influenza A(H1N1pdm09) viruses from Kazakhstan; Supplementary Figure S2. Phylogenetic subtrees and geographic composition of clusters containing influenza A(H3N2) viruses from Kazakhstan; Supplementary Figure S3. Phylogenetic subtrees and geographic composition of clusters containing influenza B(Victoria) viruses from Kazakhstan; Supplementary Figure S4. Phylogenetic subtrees and geographic composition of clusters containing influenza B(Yamagata) viruses from Kazakhstan.
